# Sustainable chitosan nanocomposite films reinforced bio silica for UV protective and active food packaging

**DOI:** 10.1186/s11671-026-04959-y

**Published:** 2026-10-22

**Authors:** Fatma N. El-Shall, Amal H. Abdel Kader, Mohamed A. Diab, Mona A. El-Sabour, Abeer M. Adel

**Affiliations:** 1 Dyeing, Printing and Auxiliary Department, National Research Centre, 33 El-Bohouth St. (Former El-Tahrir St.), Dokki, P.O. 12622, Giza, Egypt; 2 Cellulose and Paper Department, National Research Centre, 33 El-Bohouth St. (Former El-Tahrir St.), Dokki, P.O. 12622, Giza, Egypt

**Keywords:** Chitosan@bio-silica, Film food packaging, Cherry tomatoes, UV-shielding, Suntest CPS

## Abstract

Chitosan was integrated with rice husk-derived bio-silica nanoparticles, including both silica-based nanocellulose (NC) and pure silica nanoparticles (SiO₂), to develop environmentally friendly active packaging films designated as CS@NC-SiO₂, valorizing agricultural waste within a circular-economy framework. The bio-silica Particles were prepared by citric-acid pretreatment followed by calcination at 600 °C. Films containing 0.25–1 wt% SiO₂, with and without NC, were systematically evaluated, and both the nanoparticles and the resulting nanocomposites were characterized by SEM, EDX, TEM, FTIR, and XRD. The optimized CS@NC–SiO₂ film, loaded with 0.75% SiO₂, exhibited marked improvements in several key properties. It recorded char residue 51% at 700 °C (compared with 39% for pure chitosan). The water contact angle increased to 94.6°, and water-vapor permeability fell to 1.146 g/(h·m·atm). it showed better mechanical properties than the pure chitosan film: tensile strength rose to 25.37 MPa, elongation at break to 27%, and Young’s modulus to 593 MPa. The same film provided strong UV protection (UPF 57.8% (3.0% UVA and 1.8% UVB transmission) and showed high antibacterial activity (98.12% inhibition of *Escherichia coli* and 61.70% inhibition of *Staphylococcus aureus.* The CPS^+^ accelerated weathering tester was used to evaluate the performance of the fabricated films under UV irradiation and to assess their impact on the shelf life and weight stability of cherry tomatoes. fabricated films under UV irradiation and to assess their impact on the shelf life and weight stability of cherry tomatoes. The results revealed that the CS@NC-SiO₂ film limited cherry tomato weight loss to 16.12% after 21 days of storage versus 31.74–71% for the other formulations, while effectively acting as UV The formulations acted as effective UV blockers and provided active antimicrobial properties, successfully preserving the quality of the packaged cherry tomatoes for up to 21 days. This work delivers sustainable, low-cost active packaging films with strong potential for food preservation.

Clinical trial number : Not applicable.

## Introduction

Tomato *(Solanum lycopersicum)* is one of the most widely grown and consumed fruits globally, owing to its ease of use and its richness in beneficial bioactive compounds [1,2]. Tomatoes belong to the climacteric fruit category and are prone to spoilage. During post-harvest storage, they persistently undergo respiration and biochemical ripening, which accelerate aging and decay [3]. Hence, packaging is fundamental to preserving postharvest tomato quality. Many strategies have been applied to retain the quality and enhance the postharvest longevity of fresh produce (particularly fruits and vegetables), such as through cold storage, controlled atmosphere packaging, and chemical preservation methods [4]. In recent years, efforts have focused on creating eco-friendlier preservation and packaging technologies to replace conventional synthetic polymer-based packaging. Bio-composite films produced from natural polymers, such as polysaccharides, proteins, and lipids from plant or animal sources, have emerged as highly effective alternatives for food preservation, owing to their renewability, biocompatibility, biodegradability, and environmental safety [5] .

Chitin, a crucial structural component of arthropod exoskeletons, gives rise to chitosan upon deacetylation. This derivative is one of the most outstanding natural polymers for biodegradable packaging films, largely because of its inherent antioxidant features and remarkable film-forming ability [6]. Chitosan films act as barriers that inhibit microbial and fungal spoilage, and they have already proven effective on numerous types of fresh fruits and vegetables [4,7]. Functionalized chitosan can be used to form a biopolymer nanocomposite film that preserves food by overlaying the product’s surface. The degradation of fresh produce is a complex, multi-mechanism biochemical process involving moisture loss, ongoing respiration, microbial spoilage, and UV-induced oxidative damage. Although most current studies on food-preservation materials emphasize mechanical strength, gas-barrier performance and antibacterial activity, the role of UV light in food spoilage has received comparatively little attention [8] Of particular relevance is the fact that ultraviolet light in the range (200–400 nm) present in sunlight promotes the rapid deterioration of key nutrients (e.g., proteins, lipids, and vitamins) within fresh produce. The consequent decline in nutritional value is accompanied by a considerable shortening of product shelf life [9]. Ultraviolet irradiation not only promotes food degradation but also heightens the risk of pathogenic contamination, for instance by *Escherichia coli* and *Listeria monocytogenes*, which are among the most common etiological agents of foodborne illness [8]. In response to the health and environmental issues associated with non‑biodegradable plastic packaging, research has increasingly focused on UV‑protective films made from biopolymers sourced from renewable natural materials, such as chitosan, cellulose, starch, gelatin, and alginate [10]. The non-toxicity, biocompatibility, and biodegradability of these biopolymers make them sustainable replacements for synthetic polymers [11,12]. However, pure chitosan films have drawbacks including poor water resistance and weak mechanical properties, greatly limiting their practical applications [13, 11]. To overcome these intrinsic limitations, improving performance generally involves either mixing with other substances or adding functional nanofillers [14,15,16]. Chitosan can form high- performance composite films by interacting with bio-polymers (e.g., starch, pectin, gelatin, and lignin) via intermolecular hydrogen bonding or ionic interactions [17, 18] .

Agro waste management plays a key role in achieving sustainable use of resources, particularly given about 35 million tons of residues generated annually [19, 20]. In the past, such wastes, including sugarcane bagasse, wheat straw, rice husks, and corn stalks, were either burned or composted. However, more recent approaches have turned toward transforming these materials into high-value products, such as nanocellulose (NC) [21]. Because nanocellulose (NC) is an abundant natural biopolymer, it can be efficiently processed into a variety of materials, including hydrogels, films, paper, and composites [22, 23, 24]. Nanocellulose (NC) is characterized by its renewability, lack of toxicity, and biodegradability, in addition to superior mechanical properties, extensive surface area, high degree of crystallinity, and low density [25, 26]. Even with these advances, there continues to be a need for cellulose-based emulsions that more thoroughly integrate antibacterial activity, moisture resistance, and other vital properties for food preservation and packaging. The application of nanocellulose (NC) in packaging is hindered by its inadequate UV-blocking capability and poor thermal stability. To address this drawback, chitosan biopolymer formulations are supplemented with nanomaterials (e.g., SiO_2_, ZnO, TiO_2_, Cu NPs, CeO_2_, CQDs, plant extracts). These additives confer better vapor barrier, UV-blocking, and antioxidant/antimicrobial properties, thus lengthening the preservation period of food products [27, 28, 29]. Porous Silica nanoparticles are highly regarded for their outstanding thermostability, large pore volume, and high surface area. These properties have made them the focus of substantial research and extremely useful for enhancing material performance, particularly in boosting the mechanical and thermal stability of biopolymer-based films. There are many ways to synthesize silica nanoparticles, for instance, thermal evaporation, chemical vapor deposition, metal‑catalyzed methods, template‑assisted techniques, electrospinning, and sol‑gel processing. Nevertheless, these techniques often come with significant drawbacks, such as the need for costly equipment and the generation of impurities that may compromise the stability and purity of the resulting nanoscale silica particles [30]. Hence, this research aims to progress a greener strategy by extracting high-purity silica nanomaterials from agro-wastes, particularly rice husks (RH) [31, 32]. The yearly production of rice husk waste (760 million tons) and rice straw waste (1.14 billion tons), each comprising 15–20% silica, makes the extraction of silica nanoparticles from this biomass a sustainable alternative [33, 34]. Research has demonstrated that rice husk ash, which can contain up to 90% silica, can be effectively converted into nano-silica with a high specific surface area using a hydrothermal process [9] .

Although chitosan nanofiller composites, rice husk-derived silica, and nanocellulose have been explored individually as potential reinforcements for biopolymer packaging, there are limited attempts that synergistically combine agro-waste-derived bio-silica both with and without nanocellulose in a single chitosan matrix. To our knowledge, the UV-protective performance of these combined films under controlled accelerated weathering has received little attention, even though UV exposure remains a major and still under examined cause of nutrient loss and increased microbial risk in fresh produce. In addition, earlier works on chitosan–silica or chitosan–nanocellulose films report mechanical, barrier, and antimicrobial properties, without checking whether those improvements actually protect a real perishable fruit. The goal of this preliminary work was to produce a chitosan@bio-silica film that acts as an effective protective barrier for maintaining the freshness of cherry tomatoes (*Solanum lycopersicum)* after harvest. For this purpose, a natural active packaging system was designed by incorporating bio-silica nanoparticles (SiO_2_ with or without NC) into a chitosan matrix. Mesoporous rice husk derived silica nanoparticles and silica-based NC were utilized to enhance the overall functionality of the packaging. A series of bio-silica nanocomposite films was produced, in which the SiO_2_ concentration was systematically adjusted. The resulting films were examined for their chemical structure, morphological characteristics, and interfacial properties. The effects of the CS@bio-silica films on key properties including mechanical strength, moisture barrier performance, hydrophobicity, UV protection, and antimicrobial activity were thoroughly evaluated. Additionally, a framework was established using the Suntest CPS+ accelerated weathering tester to assess the films’ performance under ultraviolet (UV) irradiation. Packaging performance tests further examined the films’ ability to preserve fruit quality, particularly their impact on shelf-life and weight stability of cherry tomatoes. Chitosan bio‑silica nanocomposite films are considered promising candidates for active packaging applications.

## Experimental

### Materials and reagents

All chemicals utilized in this investigation were of high analytical purity. Chitosan (CS) with a degree of deacetylation between 90 and 95% and a molecular weight of 400,000 was obtained from Oxford Laboratory, India. The waste bleached pulp was kindly sourced from the white water of the Quena Paper Mill in Quena, Egypt. This white water, a papermaking byproduct, along with the waste pulp, was analyzed for chemical composition following Tappi standard methods. The results were as follows: α-cellulose, 71.04 ± 0.01% (T 203 cm-99); lignin, ≥ 2.00% (T 222 om-88); hemicellulose, 14 ± 0.10% (T 223 cm-84); and ash, 8.77 ± 0.02% (T 211).

### Extraction of bio-silica nanoparticles

#### Extraction silica-based nanocellulose particles (NC)

Rice agro-waste pulp was suspended in 64% (w/w) sulfuric acid at a solid-to-liquid ratio of 1:20 (w/w) and magnetically stirred in a heated water bath at 45 °C for 25–30 min. Hydrolysis was quenched by the addition of ice-cold water. The resulting suspension was washed repeatedly with distilled water, centrifuged at 6000 rpm for 15 min, and dialyzed against distilled water for approximately one week until neutral pH was reached. The nanocellulose (NC) suspension (yield 67.23 ± 1.62%, crystallinity index CrI = 82%) was sonicated for 10 min prior to use.

#### Pure silica nanostructure separation from rice husk

Rice husks (RHs) were obtained from a local rice mill in Egypt. RHs were first rinsed with tap water to get rid of dirt and soluble impurities, then air-dried at ambient temperature for 48 h, and finally ground into a fine powder. The cleaned, dried husks were kept in polyethylene bags at ambient temperature until further use. Rice husks served as an inexpensive and plentiful source of biogenic silica nanoparticles (SiO_2_), enabling the production of nanoparticles with controlled morphological characteristics, crystalline phase, and purity level. This result was achieved through calcination of RHs in a muffle furnace heated to 600 °C, following a pretreatment with 1 M citric acid at 120 °C for 2 h. After calcination, the silica in the resulting rice husk ash was extracted as sodium silicate using 2 M NaOH. Then, 2 M hydrochloric acid was added dropwise to the sodium silicate solution until the pH was adjusted to 7. The proposed alkali/acid reaction mechanism is as follows: First, RHA is dissolved in sodium hydroxide to form sodium silicate (Eq. 1). Second, precipitation occurs with hydrochloric acid (Eq. 2):

1 $$2\mathrm{NaOH}+{x}\mathrm{SiO}_{3}\to\:\mathrm{Na}_{2}\mathrm{SiO}_{3}+\mathrm{H}_{2}\mathrm{O}$$

2 $$\mathrm{Na}_{2}\mathrm{SiO}_{3}+2\mathrm{HCl}\to\:\mathrm{SiO}_{2}\left({gel}\right)\downarrow\:+2\mathrm{NaCl}+\mathrm{H}_{2}\mathrm{O}$$

The extended three‑dimensional Si–O–Si network was generated by alkaline treatment, after which silanol groups (R_3_Si–OH) were solubilized with hydrochloric acid and underwent subsequent condensation [35].

### Preparation and incorporation of nanoparticles in chitosan films

Chitosan (CS)-based bio-nanocomposite films incorporating nanocellulose (NC) were fabricated using the solution casting technique. A CS solution was prepared by mechanically stirring a 2% (w/v) CS mixture in 1% acetic acid at 50 °C for 3 h. Any undissolved material or impurities were removed by filtration through a nylon membrane. Films were then produced by blending the CS solution with 7% (w/w NC) fractions; this specific NC loading was chosen based on prior findings from a study involving different NC polymorph concentrations in CS films [14]. The composite mixtures were homogenized at 500 rpm for 5 min, then 30% (w/w) glycerol was added as a plasticizer, and stirring continued at 50 °C for another hour. To enhance the even distribution and minimize agglomeration of SiO_2_ nanoparticles within the chitosan matrix, their surface was modified by suspending the nanoparticles in 50 mL of deionized water with 15% (w/w) sodium dodecyl sulfate, followed by magnetic stirring at 40 °C for 30 min. Films were subsequently prepared by mixing the CS solution with various concentrations (0.25–1.5% SiO_2_ nanoparticles) of fractions, either with or without NC. After vacuum degassing to eliminate trapped air bubbles, the homogeneous mixtures were cast onto PTFE plates. The cast films were then dried at 35 °C for 18 h. The resulting films had a thickness of approximately 70 μm. Prior to testing, the films were conditioned for 2 days at 25 °C and 51% relative humidity in a desiccator using a saturated magnesium nitrate solution. The sample codes used are CS, CS@NC, NC@SiO_2_, and CS@NC-SiO_2_.

### Characterization of bio-silica nanoparticles, and chitosan films

#### Morphological examination

##### Transmission electron microscopy (TEM) 

The morphology and particle size of the extracted nanoparticles were characterized using a Transmission Electron Microscope (TEM, model JEM-1230, JEOL, Japan) operating at an accelerating voltage of 120 kV. The instrument was used with a magnification of 600,000× and a resolution of 0.2 nm. Sample preparation involved depositing a small droplet of the nanoparticle dispersion onto a carbon-coated copper grid, followed by air drying before insertion into the TEM chamber.

##### Scanning electron microscopy with energy dispersive X-ray spectroscopy (SEM/EDX)

The surface morphology of the fabricated films was examined using SEM/EDX (model JSM 6360LV, JEOL/Noran, USA). To minimize beam-induced damage, the samples were positioned at the maximum possible distance from the electron beam source. Prior to analysis, the films were sputter-coated with a thin layer of gold at a low deposition rate and subsequently cooled. Micrographs were acquired at an accelerating voltage of 20 kV.

#### Functional chemistry analysis

##### Fourier-transform infrared spectroscopy (FT-IR)

The chemical structure and functional groups of the prepared films were analyzed using an FTIR-8400 S spectrometer (Shimadzu Europe Co., Kyoto, Japan) via transmission mode. Spectra were recorded over a wavenumber range of 500–4000 cm⁻¹ at a resolution of 4 cm⁻¹ with 32 scans. 

##### X-ray diffraction (XRD)

X-ray diffraction patterns of the films were obtained at room temperature using a Philips diffractometer (Model PW 1390, Japan) equipped with nickel-filtered Cu Kα radiation (λ = 1.5404 Å).

##### Thermal analysis

Thermogravimetric analysis (TGA) was performed using a PerkinElmer instrument (Waltham, Massachusetts, USA) under a nitrogen atmosphere at a heating rate of 10 °C/min. Differential scanning calorimetry (DSC) was also conducted under the same nitrogen atmosphere over a temperature range of 25–600 °C at a heating rate of 10 °C/min.

### Mechanical measurements, water vapor permeability (WVP), and water contact angle (WCA) analysis

#### Tensile properties

Tensile testing was conducted according to the TAPPI T494–06 standard using a universal testing machine (Model LR10K; Lloyd Instruments, Fareham, UK) equipped with a 100 N load cell. Tests were performed at a constant crosshead speed of 2.5 cm/min. The key parameters measured included tensile strength (MPa), Young’s modulus (MPa), elongation at break (mm), and stiffness (N/m). Each test was performed in triplicate, and results are reported as mean values ± standard deviation.

#### Water vapor permeability (WVP)

Water vapor permeability of the chitosan bio-nanocomposite films was determined according to the ASTM E96/E96M-10 standard. Prior to testing, the films were conditioned at 25 °C and 50% relative humidity for 24 h. The conditioned films were then used to seal aluminum cups containing 5 g of anhydrous calcium chloride, with the film’s uncoated side facing the external environment. WVP values were calculated using Eq. (3)

3 $${WVP}={g}\times{h}/{t}\times{A}\times{\Delta}{P}$$

where *(g/t)* is the slope of the weight gain *(g)* versus time (hours) plot, *h* is the average film thickness, *A* is the exposed permeation area, and *ΔP* is the difference in partial water vapor pressure (≈ 2385 Pa) between the external environment (75% RH, maintained using saturated sodium chloride solution) and the inside of the cup (0% RH). All WVP values are reported as the average of three independent measurements.

#### Water contact angle measurement

The surface hydrophobicity of the prepared chitosan films was evaluated by measuring the water contact angle using an OCA-15EC instrument (Dataphysics GmbH, Germany) with its dedicated software. A 10 µL droplet of triple-distilled water was placed on the film surface at three different locations, and the contact angle was recorded.

### Porosity analysis

The specific surface area of the samples was determined using a Quantachrome Touch Win™ instrument according to the Brunauer–Emmett–Teller (BET) method. Approximately 200 mg of each film was cut into small pieces (1 mm × 1 mm), placed in a glass cell, and degassed at 60 °C under vacuum (50 mTorr) for 4 h. Pore volume and average pore diameter were calculated using the Barrett–Joyner–Halenda (BJH) method.

### UV performance tests

#### UV-Vis transmittance measurement

The optical transmittance of the fabricated films was measured across the wavelength range of 200–800 nm using a JASCO V-730 double-beam UV-Vis/NIR spectrophotometer (JASCO Corporation, Tokyo, Japan).

#### Ultraviolet protection factor determination: 

The ultraviolet radiation (UVR) transmittance and ultraviolet protection factor (UPF) of the films were evaluated in accordance with the Australian/New Zealand Standard AS/NZS 4399:1996 using a Shimadzu UV-3101 PC UV-Vis-NIR spectrophotometer (Shimadzu Corporation, Japan).

### Antibacterial activity determination

The antibacterial activity of the chitosan@bio-silica films was evaluated using the broth dilution method, with bacterial growth monitored by measuring optical density at 600 nm. The tested strains *Bacillus cereus* ATCC 33,018, *Staphylococcus aureus* ATCC 12,600, *Pseudomonas aeruginosa* ATCC 33,354, and *Escherichia coli* O157:H7 ATCC 6933 were first cultured on nutrient agar plates for 18 h at 37 °C. A single colony of each strain was then transferred into nutrient broth and incubated overnight at 37 °C. Subsequently, the overnight cultures were inoculated into 3 mL of fresh nutrient broth, and approximately 72.5 mg of the composite film material was added to each tube. After 24 h of incubation at 37 °C, the absorbance was measured at 600 nm. The percentage of bacterial growth inhibition was calculated using the standard formula.

$${Growth}\,{Reduction}=\frac{OD_{c}-{OD}_{s}}{OD_{c}}\times100$$

Where, OD_c_ = Optical density of the control measured at 600 nm, and OD_s_ = Optical density of the sample measured at 600 nm.

### Application of fabricated films as UV blocker for cherry tomato packaging

Cherry tomatoes *(Solanum lycopersicum)* were bought from agricultural local markets. Fruits with uniform size, color, and appearance, and free from visible mechanical damage, disease symptoms, or defects were carefully selected for the experiments. Cherry Tomatoes were washed with a 2% acetic acid solution, thoroughly dried, and individually wrapped in the tested film sheets. The edges of the film were sealed with transparent tape to ensure complete enclosure. The wrapped samples were then exposed to the Suntest CPS^+^ accelerated weathering xenon tester chamber for a total of 10.5 h, delivered at a rate of 30 min per day over 21 days. According to the Suntest CPS^+^ tester (a very high UV irradiance, typically around 60–80 W/m² in the 300–400 nm range) specifications, 60 min of exposure to its xenon light source is equivalent to 540 min (9 h) of real-world outdoor sunlight exposure. This irradiation regimen is corresponding to a daily exposure equivalent to 4.5 h of natural sunlight for 21 consecutive days. Following the irradiation period, all samples were stored in a refrigerator at 4°C controlled temperature. Sample weight was recorded every 7 days throughout the 21 days of the storage period to determine the weight loss percentage using the following equation:

4 $$Weight\:loss\:\left({\%}\right)={m}_{\mathrm{o}}-{m}_{1}/{m}_{\mathrm{o}}\times100\%$$

where *m*_o_ is the initial weight of fresh strawberries, and *m*_1_ is the weight of cherry tomatoes after storage for a specific time.

### Statistical analysis

All the experiments were performed in triplicates and the data obtained were presented as the mean ± standard deviation. Statistical techniques based on the Analysis of Variance (ANOVA) are used to examine all the findings and observations, presented as mean standard deviation.

## Results and discussion

### Morphological study

#### Bio-silica nanoparticles’ structure

**Fig. 1:**
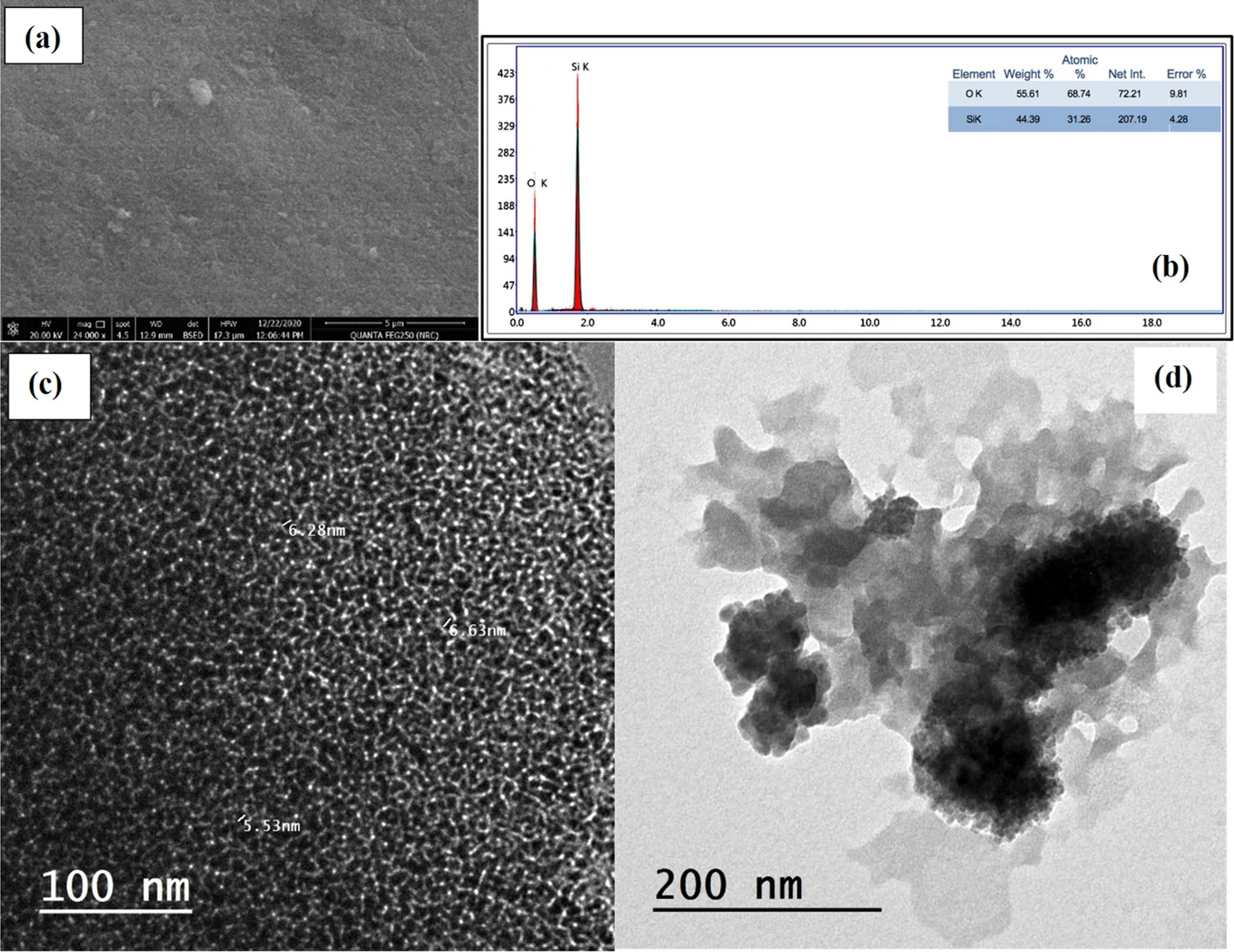
SEM photo **a**; EDX analysis **b** for bio-silica; TEM micrograph **c** for SiO2np; and silica-based NC **d**

Citric acid was selected as the pretreatment agent because it is a mild triprotic acid that also functions as a metal-chelating agent. This dual role allows it to selectively remove alkali and alkaline-earth impurities (K, Na, Ca) without the aggressive matrix degradation that strong mineral acids typically cause. Although HCl leaching can produce silica of higher purity, it generates corrosive and non-biodegradable waste [36,37]. Citric acid, by contrast, is inexpensive, biodegradable, and produces far fewer hazardous by-products [38]. Alkali pretreatment with NaOH is effective at delignification but tends to dissolve excessive biomass and can cause significant sugar degradation. Moreover, NaOH pretreatment generates saponifiable waste streams and requires extensive washing. Comparative screening studies have shown that acids generally perform better than alkalis for rice husk pretreatment [32,39]. The gentle, chelation-based cleaning is clearly reflected in the nanoparticles that were obtained. SEM images of the nano-silica surface (Fig. 1a) show an amorphous morphology with nanoscale roughness, consistent with a pretreatment that strips away metallic impurities without the more aggressive structural alteration associated with mineral-acid leaching. EDX analysis (Fig. 1b) confirmed a silicon content of 44.39%, indicating that he isolated material consists mainly of silicon and oxygen and possesses a high degree of purity [9,40]. While this purity is lower than the 91–99% values reported for HCl-eached rice-husk silica, it represents a deliberate trade-off: the process prioritizes a safer, biodegradable route over the highest possible purity.

TEM examination of the two bio-silica samples (Fig. 1c, d) further supports this interpretation. The cellulose nanocrystals appeared spherical, while the mesoporous silica nanoparticles displayed a regular, uniform shape with only limited agglomeration. Because citric acid preferentially chelates surface-bound metals rather than uniformly corroding the silica framework, the native mesoporous architecture inherited from the rice husk’s cellular structure is largely preserved instead of being collapsed or fragmented, as often occurs with harsher mineral-acid treatments.

#### Chitosan bio-nanocomposite film structure

**Fig. 2:**
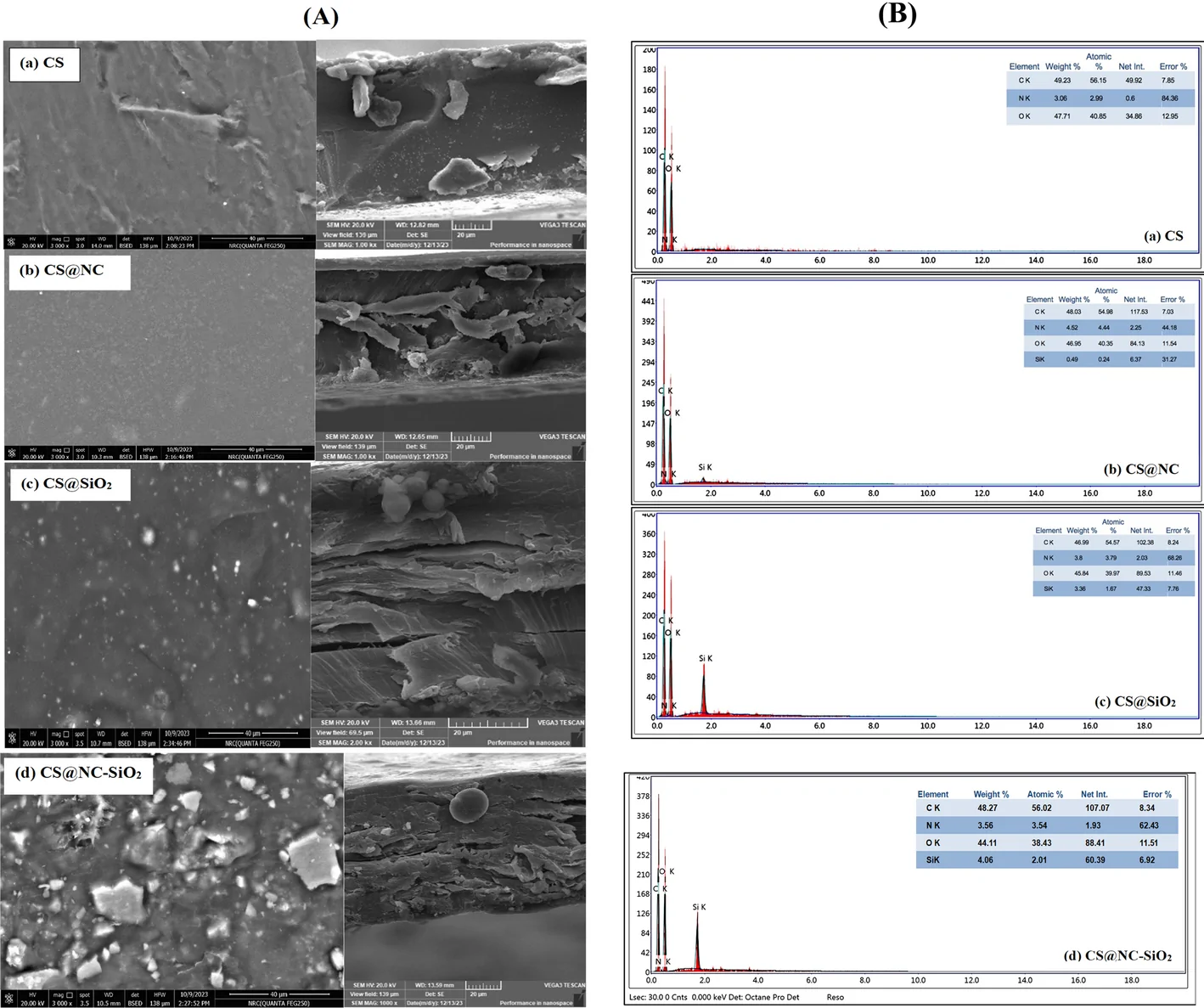
**A**. SEM images (surface left, cross-section right) and **B**. EDX spectra of chitosan hybrid films: **a** CS, **b** CS@NC, **c** CS@SiO_2_, and **d** CS@NC‑SiO_2_

Figure 2A(a-d) displays the surface and cross-sectional microstructures of the CS, CS@NC, CS@SiO_2_, and CS@NC-SiO_2_ nanocomposite films, which are loaded with silica-based NC and/or SiO_2_ nanoparticles. The pristine CS film exhibits a dense, smooth, and uninterrupted surface with no surface irregularities. In contrast, the addition of NC with or without SiO_2_ nanoparticles leads to diverse structural features. The CS@NC bio-composite film has a uniform and smooth surface, free from macroscopic bubbles, cracks, or pores. Unlike the smooth and homogeneous surface of the pristine CS film (Fig. 2A(a, b)), the CS@NC and CS@SiO_2_ bio-composites display small flakes or dots on their surfaces (Figs. 2A(b, c)), indicating that the nanoparticles are well distributed within the polymer matrix and have good miscibility and compatibility with chitosan. However, the CS@NC-SiO_2_ composite shows non‑uniform dispersion, with pronounced wrinkles and extensive agglomerates covering the film surface (Fig. 2A(d)). The inclusion of NC and/or SiO₂ nanoparticles results in a more evenly distributed composite due to the formation of numerous hydrogen bonds between the nanoparticles and the chitosan matrix, leading to a cross‑linked network structure between the nanoparticles and the polymer [41]. As seen in Fig. 2A(d), the CS@NC-SiO_2_ composite film has an uneven surface without clear pores, yet particle aggregation is present. This results from the high concentration of nano-SiO_2_. Besides forming intermolecular hydrogen bonds with chitosan and nanocellulose, some unbound silanol groups remain, which have high surface energy and tend to aggregate with each other. The cross-sectional microstructure (Fig. 2A) shows a somewhat homogeneous but rough texture in both the CS@SiO_2_ and CS@NC-SiO_2_ nanocomposites. This roughness is attributed to the tight deposition of SiO_2_ onto the chitosan surface, which creates a non-uniform layer of silica nanoparticles [42, 43] .

In chitosan/SiO_2_ bio-composite films for packaging applications, Energy Dispersive X-ray Spectroscopy (EDX) is used to verify the existence and dispersion of bio-silica nanoparticles throughout the chitosan matrix. The elemental composition of the CS and modified CS nanocomposite films was analyzed using EDAX (Fig. 2B(a–d)). Figure 2B(c, d) reveals a strong silica peak, demonstrating that the CS@SiO_2_ and CS@NC-SiO_2_ hybrid films contain a significantly higher amount of silica.

### Structural properties of chitosan films

**Fig. 3:**
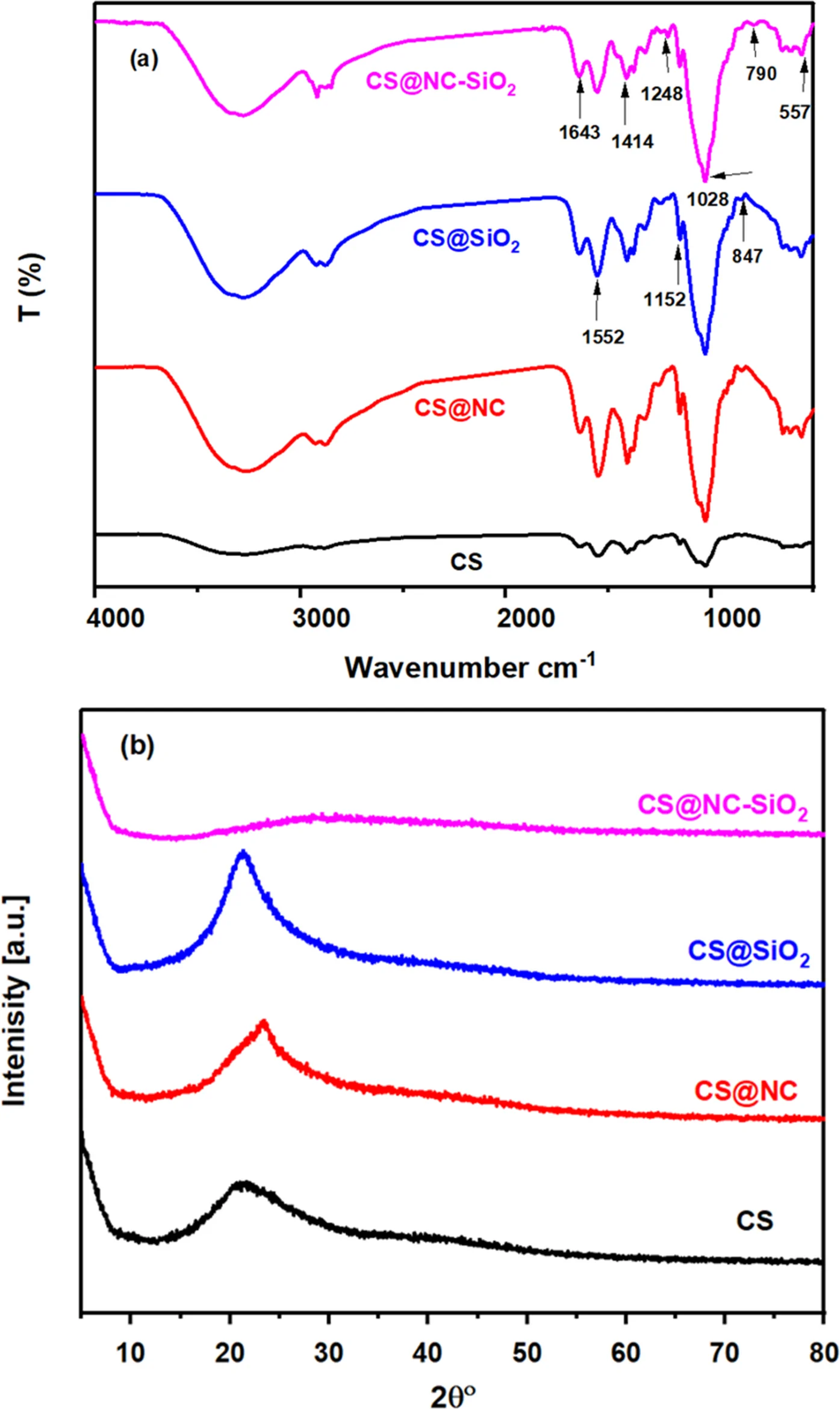
FTIR **a**; and XRD profiles **b** for chitosan hybrids films CS **a**, CS@NC **b**, CS@SiO_2_ **c**, and CS@NC-SiO_2_ **d**

Figure 3(a) presents the FTIR spectra recorded to investigate structural differences between the chitosan‑based bio‑silica composite films with and without NC. The pure and modified chitosan films displayed intense absorption bands at 3340 cm⁻¹ and 3290 cm⁻¹, respectively, assigned to the stretching vibrations of –OH and the –NH_2_ groups in chitosan. The reduced intensity of the absorption peak at 3290 cm⁻¹ for the modified films resulted from hydrogen bonding with the nanoparticles (SiO_2_ with or without NC). Specifically, the hydroxyl groups of nanocellulose and the silanol –OH groups of nano-silica interacted with the chitosan films, thereby improving compatibility between the components [41,44]. The assigned absorption at 2890, 2919, and 2866 cm⁻¹ were attributed to C–H stretching vibrations in both the neat and hybrid chitosan films. The band at 1643 cm⁻¹ was likely related to C = O stretching, denoting the C = O stretching vibration in the –CONH_2_ bonds. Additionally, the amide II band (N–H bending vibration) appears at 1552 cm⁻¹, while the band at 1414 cm⁻¹ is attributed to the bending and deformation absorptions of –CH₂ and –CH₃ groups. For the (CS, CS@NC, CS@SiO_2_, and CS@NC-SiO_2_) nanocomposite films, the characteristic bands at 1152 and 1028 cm⁻¹ in Fig. 3(a) for unaltered and altered chitosan with nanoparticles (SiO_2_ with/without NC) typically correspond to C–O stretching vibrations [45,46] .

These are strong indicators of the presence of β-(1,4)-glycosidic linkages characteristic of these polysaccharide structures. The presence of SiO_2_ enhances the peak intensity related to the Si–O–Si bond, typically in the region of 1000–1250 cm⁻¹, which often overlaps with the chitosan peaks in this range [47]. Furthermore, the peaks at 847 cm⁻¹ (attributed to the SiO_4_ tetrahedron) and 557 cm⁻¹ (assigned to O–Si–O deformation) showed increased intensity in the CS@NC, CS@SiO_2_, and CS@NC-SiO_2_ samples. Additionally, a clear peak at 790 cm⁻¹ observed in the CS@NC-SiO_2_ composite film verified that SiO_2_ nanoparticles were successfully incorporated onto the film surface [42]. The bands observed at about 790 cm⁻¹ and 1248 cm⁻¹ represent the symmetric bending vibrations of Si–CH_3_ groups in the modified chitosan films. This observation confirms that bio-silica (SiO_2_) incorporating nanocellulose (NC) has been successfully crosslinked with the chitosan matrix [9] .

Figure 3(b) presents the XRD patterns of CS, CS@NC, CS@SiO_2_, and CS@NC-SiO_2_. CS and CS@NC show diffraction peaks at 2θ = 21° and 23°, respectively, indicating that the chitosan structures are partially semi-crystalline or amorphous. The crystallinity observed in chitosan results from hydrogen bonding between its molecular chains; these bonds influence the crystallization behavior, and the crystalline form varies with the molecular conformation of chitosan [47]. The diffraction pattern of CS@SiO_2_ shows no polycrystalline peak except for a broad one centered at 2θ = 23°, indicating that SiO_2_ has an amorphous/crystalline structure incorporated within the chitosan structure. Nano-silica that undergoes surface treatment bears numerous –Si–OH groups, enabling strong hydrogen bonding with chitosan molecules. These bonds influence molecular inter-chain interactions and the mobility of chitosan to a certain extent, thereby hindering the crystallization process of chitosan. In the XRD patterns of CS@NC-SiO_2_, the diffraction peaks at 2θ = 21° and 23° disappear. The synergistic effect of NC and silica nanoparticles partially disrupts the hydrogen bonds between chitosan molecules, thus affecting the semi-crystalline structure of the chitosan matrix and offering abundant growth sites for the formation of an amorphous structure [48, 49] .

#### Molecular interaction mechanisms in the CS@NC-SiO₂ system

The behaviour of the CS@NC-SiO₂ nanocomposite films can be described by three intertwined interactions: hydrogen bonding, electrostatic attraction, and interfacial siloxane condensation. Hydrogen bonding Chitosan –OH/–NH_2_ groups establish hydrogen bonding with NC hydroxyl groups and SiO_2_ hydroxyl (silanol) groups. This is confirmed by FTIR (Fig. 3a), a shifted in –OH/–NH_2_ stretching band is observed in the composite films, suggesting these moieties are involved in intermolecular bonding rather than being free [50]. This hydrogen-bonded network connects the organic (chitosan/NC) and inorganic (SiO_2_) components, accounting for the enhanced interfacial compatibility (SEM, Fig. 2A (b, c)) as well as the tensile strength improvement. Electrostatic forces, under the weak acidic casting environment (1% acetic acid), chitosan’s –NH₂ groups are partially protonated into –NH₃⁺, which results in the polymer of a positive charge in net. NC (sulfate esters) and SiO₂ (SiO⁻) are negatively charged at the surface, allowing electrostatic interaction with chitosan [51]. This charge complementarity leads to an enhanced dispersion of nanoparticles (Fig. 2A (b, c)) at intermediate loadings. The FTIR bands for Si–CH₃/Si–O–Si as well as SiO₄ and O–Si–O are related to silanol condensation within the chitosan interface, suggesting the formation of a covalently bonded Si–O–Si network rather than simple hydrogen bonding [52]. This is in line with diminishing porosity (Table 2), and accounts for the concentration-dependent decrease in water vapor permeability, regardless of hydrophobicity enhancement observed from contact-angle measurement (Sect.  3.4). Hydrogen bonding is the primary organic–inorganic adhesion; electrostatic interaction controls the dispersion and siloxane condensation introduces physical cross-linking that enhances barrier density.

### Thermal behavior of chitosan films

Thermogravimetric analysis (TGA) is a common method used for studying how materials lose weight under controlled heating. It reliably evaluates thermal properties, phase transitions, and component interactions within chitosan biopolymer blends. For packaging materials, heat resistance is crucial to preserve structural integrity and functionality during food storage and transportation [53] .

**Fig. 4:**
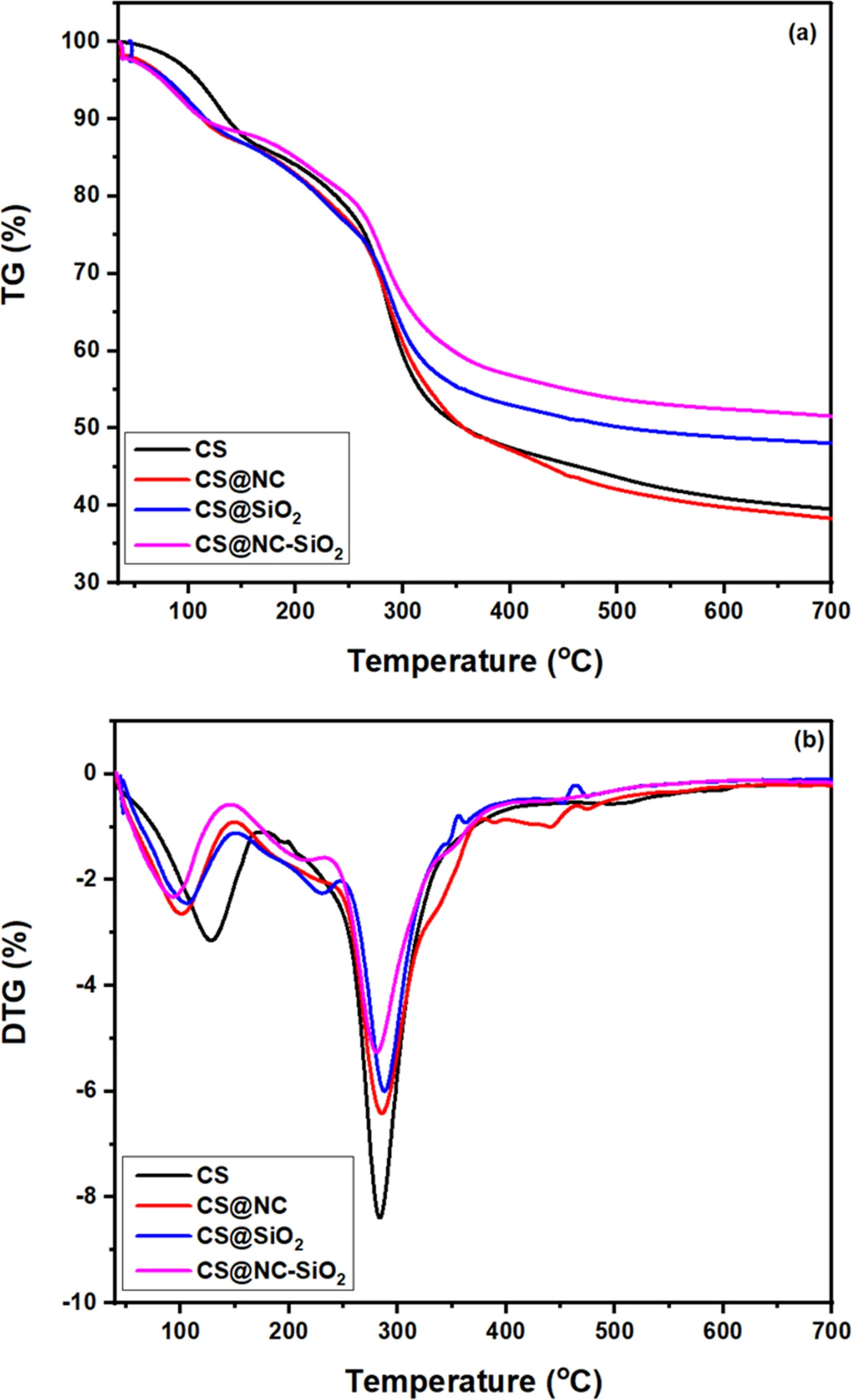
TGA **a**; and DTG measurements **b** for chitosan hybrids films CS **a**, CS@NC **b**, CS@SiO_2_ **c**, and CS@NC-SiO_2_ **d**

A lower rate of sample decomposition generally indicates higher thermal stability at a specific temperature. Figure 4(a) displays the TGA profiles of the films. The TGA data were analyzed to determine the thermal stability of CS, CS@NC, CS@SiO_2_, and CS@NC-SiO_2_ composite films, an important factor for their practical applications. As shown in Fig. 4(a), thermal degradation occurred in three stages. The first stage involved an initial mass loss of roughly 8%, 9%, 7%, and 7% near 100 °C for the CS, CS@NC, CS@SiO_2_, and CS@NC-SiO_2_ films, respectively, mainly due to evaporation of physically absorbed water evaporating. The second stage took place at 283 °C, 285 °C, 287 °C, and 280 °C, with corresponding weight losses of 33.54%, 33.93%, 33.17%, and 27.66%, largely caused by the removal of volatile organic matter from the chitosan chains. The third stage occurred at 389 °C, 373 °C, 340 °C, and 393 °C, with weight losses of 60.40%, 61.52%, 52.12%, and 48.23%, respectively, resulting from the gradual carbonization of the polymer backbones, leading to sustained weight loss. All chitosan biopolymer samples reached nearly complete degradation at about 696 °C, mainly from the breaking of C–C and C = C bonds in the chitosan chains and the release of small hydrocarbon molecules (roughly 500–580 °C). Metals, pigments, fillers, and additives can greatly modify the mechanism of degradation, the resulting decomposition products, and the material’s overall thermal stability [54, 42]. When incorporated as fillers, NC and SiO_2_ nanoparticles appear to inhibit the emission of volatile organic compounds during thermal decomposition, thereby acting as a mass transfer barrier. From Table 1, all modified samples investigated here produced more char and displayed greater thermal stability than the unmodified CS film. According to Table 1, at approximately 700 °C, CS@NC-SiO_2_ exhibited the highest char yield (51% compared to 39% for the CS sample). The higher char yields observed for CS@SiO_2_ and CS@NC‑SiO_2_ reflect improved thermal stability, which correlates with the addition of silica and silica‑containing nanocellulose nanoparticles. These findings are relevant to our study, as they illustrate that the composite films achieve adequate thermal insulation for practical applications, thereby addressing a critical performance requirement for packaging or surface coating materials.

As illustrated in Fig. 4(b), the DTG curves showed that both the unmodified and modified films underwent two stages of thermal degradation. For neat CS, CS@NC, CS@SiO_2_, and CS@NC‑SiO_2_, the first decomposition peak temperatures were 127 °C, 98 °C, 102 °C, and 92 °C, and the second decomposition temperatures were 283 °C, 286 °C, 288 °C, and 282 °C, respectively [55]. The findings reveal that filler incorporation enhances chitosan’s thermal stability and structural stabilization, and the thermogravimetric data suggest that the resulting nanocomposite is viable for UV-protective packaging applications.

**Table 1:** TGA and DTA data for fabricated chitosan films

Sample	Temperature range (^o^C)	T_i_ (^o^C)	T_f (_^o^C)	DTA peak (^o^C)	Mass loss (%)	RC* (Wt.%)
CS	25–138 189–389 390–696	73 253 -	166 322 -	127 283 -	8.22 33.54 60.40	- 66.46 39.60
CS@NC	38–148 232–373 374–696	38 254 -	140 335 -	98 286 -	8.46 33.93 61.52	- 66.07 38.48
CS@SiO_2_	44–149 187–340 341–697	41 260 -	143 321 -	102 288 -	9.22 33.17 52.12	- 66.83 47.88
CS@NC-SiO_2_	35–143 147–393 394–697	41 245 -	130 322 -	92 282 -	7.71 27.66 48.23	- 72.34 51.77

RC* (wt%) is the residual char percent at temperature corresponding to constant weight loss

### Mechanical properties, water vapor permeability, and water contact angle of chitosan films

**Fig. 5:**
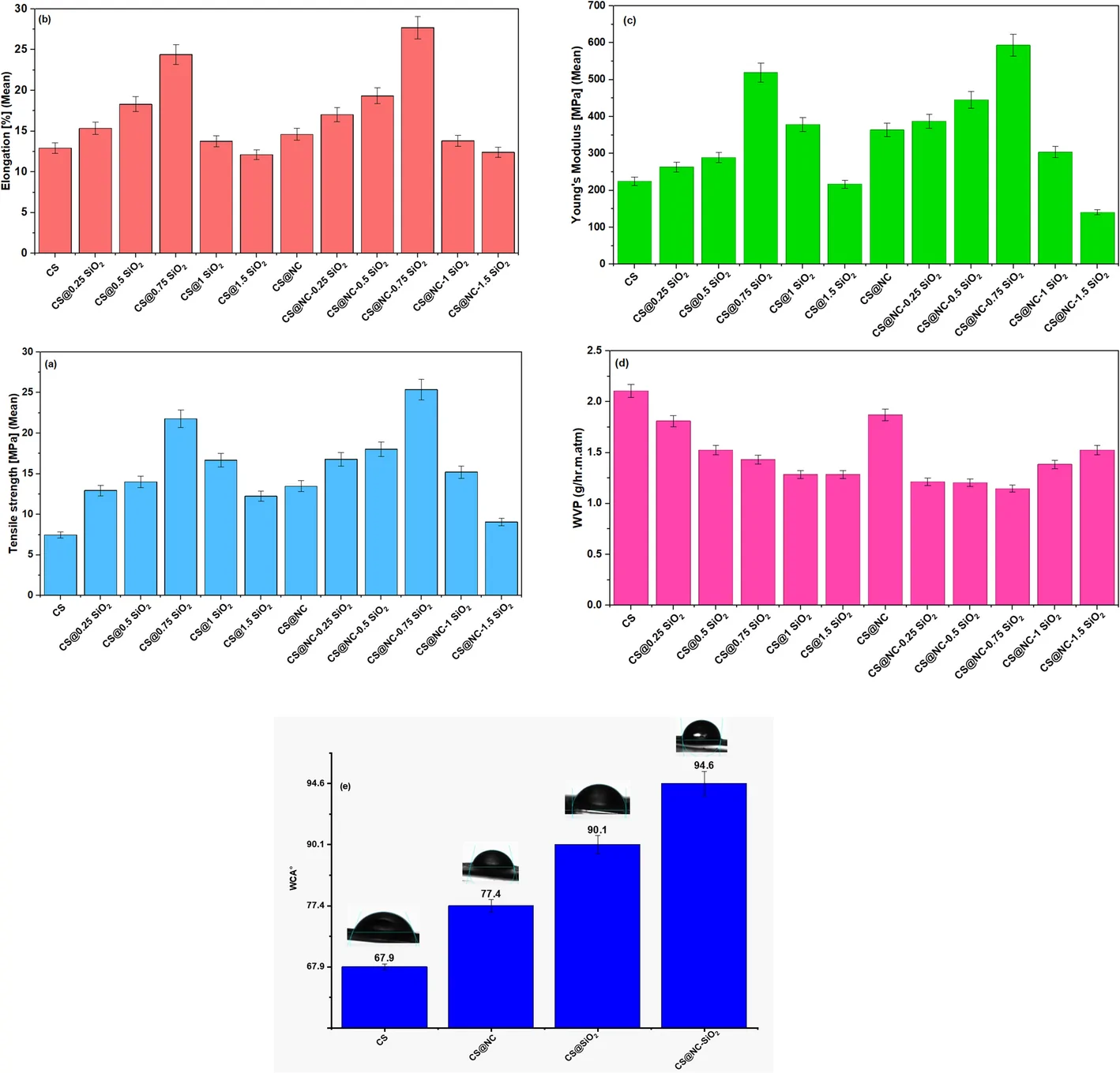
Mechanical strength and barrier properties for chitosan hybrids films, tensile strength **a**, Elongation **b**, Young’s modulus **c**, WVP **d**, and WCA **e**

Tensile strength (TS) measures a film’s ability to resist applied stress, whereas elongation at break (EB) quantifies how much the material stretches before breaking. Both properties are essential for packaging, as they protect the food from mechanical damage, preserving its integrity during storage and transportation. The mechanical behavior of the film is largely determined by intra- and intermolecular bonds between the biopolymer and filler particles. Such bonds are influenced by the orientation and packing of molecular chains in the biopolymer composite matrix; these characteristics are often improved by incorporating blended films [11]. The tensile strength, elongation at break, and Young’s modulus of nanocellulose reinforced CS films were shown in Fig. 5(a–c). In general, the CS@SiO₂ and CS@NC-SiO₂ films showed better mechanical properties than the pure CS film, especially at SiO₂ loadings of 0.25–1%. he tensile strength increased to 21.78 MPa and 25.37 MPa for CS@0.75%Si₂ and CS@NC-0.75%Si₂, respectively along with that elongation at break to 24% ad 27% ad the Young’s modulus to 518 MPa and 593 MPa. At the same SiO₂ content (0.25– 0.75%),the CS@NC and CS@NC-SiO₂ films were enhanced to the corresponding CS@ SiO₂ ones. This improvement is related to the nanoparticle’s irregular shape which facilitates mechanical interlocking with chitosan matrix and polar-polar interaction among surface sulfate groups of nanocellulose and chitosan chains [56]. This mechanical improvement is consistent with the morphological observation in Sect.  3.1.1 and 3.1.2. The extracted SiO_2_ NPs have a uniform mesoporous morphology and the silica-based NC particles are spherical (both of these features facilitate efficient stress transfer at the filler matrix interface) as can be viewed in TEM images (Fig. 1c, d). Accordingly, cross-sectional SEM images of CS@SiO_2_ and CS@NC films (Fig. 2A (b, c)) reveal the presence of fine, dispersed particles distributed on a homogeneous surface, suggesting a favorable dispersion and interfacial adhesion free of stress concentrating imperfection. The cross-sectional SEM images show that the CS@SiO_2_ and CS@NC-SiO_2_ films exhibit a rough but continuous surface (Fig. 2A), which is well consistent with the tight conformal contact and strong interfacial hydrogen bonding that enables the transfer of loads from the chitosan matrix to the rigid silica filler. For comparison, the pure CS film displays a uniform flat, filler-free surface (Fig. 2A(a)). The CS@NC-0.75%SiO2 showed the highest tensile strength and modulus (25.37 MPa and 593 MPa, respectively), which were higher than those of CS@0.75%SiO2 (21.78 MPa and 518 MPa). Indeed, such combined action of spherical NC particles that provide mechanical interlocking and co-dispersed SiO₂ nanoparticles that restrict of polymer-chain mobility leads to a synergistic effect due to which densified cross-section morphology of hybrid film can be observed as well as enhanced and more uniform silica signal in EDX maps (Fig. 2B (c, d)) [57]. The strengthening effect is dependent on the concentration and diminishes at higher concentrations. CS@1.5%SiO₂ showed the minimum tensile strength, which is related to particle agglomeration and surface corrugation as seen in Fig. 2A(d). These agglomerates behave like stress concentrators and interfere with uniform load transfer, which leads to the observed non-monotonic mechanical behavior.

The film’s mechanical reinforcement is likely due to the abundance of cross-linking functional groups and the inclusion of glycerol. Adding SiO_2_ improved mechanical properties by restricting polymer chain mobility, as SiO_2_ particles occupy interchain spaces in the polymer blend. This is enabled by strong interfacial interactions between the high-surface-area SiO_2_, NC, and polymer backbone, along with intra- and intermolecular hydrogen bonding to polar hydroxyl groups on both the filler nanoparticles and the chitosan matrix [58]. Enhanced interfacial adhesion among different functional groups likely produced stronger interchain connections, resulting in films with greater resistance to stress. However, the CS bio-composite film loaded with 1.5% SiO_2_ nanoparticles showed the lowest mechanical strength among all samples, including the control. Two main reasons explain this decline. First, the hygroscopic nature of the nanoparticles (whether SiO_2_ alone or combined with NC) causes them to absorb more water into the film matrix, thereby lowering tensile strength. Second, at higher bio-silica concentrations, the particles tend to aggregate and disperse unevenly. This agglomeration diminishes the reinforcing effect within the polymer matrix [59].

Hydrophobic films not only prevent external moisture from compromising food products but also hinder microbial growth and reproduction, making them more effective for food preservation. The water vapor permeability (WVP) of the modified films was measured by determining the volume of water moving through the CS matrix per unit area and time under specific conditions. As shown in Fig. 5(d), the water vapor permeability of CS films treated with bio-silica gradually decreased with increasing SiO_2_ content, compared to the neat CS film. This behavior might be due to changes in the immobilized film structure, which inhibited water from penetrating the CS web. In comparison between CS@SiO_2_ and CS@NC-SiO_2_, CS@NC-0.75%SiO_2_ exhibited higher hydrophobicity (lower WVP), as shown in Fig. 5(d). Films containing neat CS showed a WVP of 2.10712 ± 0.06321 g/(hr·m·atm), which decreased significantly after mixing with SiO_2_ with or without NC nanoparticles. Among the composite films, those containing CS@NC-0.75%SiO_2_ displayed the lowest WVP of 1.1462 ± 0.03439 g/(hr·m·atm). The reduced WVP of the composite films results from the more tortuous diffusion path for water vapor, generated by the impermeable bio‑silica nanoparticles across their varying concentrations. Furthermore, the water vapor permeability of the chitosan blend films depends on multiple factors, including the type of biopolymer, the compatibility between the biopolymer matrix and the bio‑silica nanoparticles (NC or SiO₂), and the filler loading. Bio-silica partially locked the CS pore matrix through the formation of inter- and intramolecular crosslinking, as displayed in the following section, resulting in lower porosity [16, 22, 60]. Furthermore, adding 1% and 1.5% SiO_2_ nanoparticles raised the water vapor permeability of the polymer films. This rise is due to the mesoporous morphology of the SiO_2_ nanoparticles and the hydrophilic character of the nanocellulose (NC), which together form channels or “bridges” that enable water vapor to pass more easily through the film [61] .

Water contact angle (WCA) measurements were used to evaluate the wettability of the chitosan@bio‑silica nanocomposite films. WCA data Fig. 5(e) show a clear trend of increasing surface hydrophobicity with the incorporation of bio‑silica nanoparticles into the chitosan (CS) matrix. Neat CS film exhibits a WCA of 67.9°, indicating a moderately hydrophilic surface, which is typical for chitosan due to its abundant hydroxyl and amino groups. CS@NC (chitosan with silica-based nanocellulose nanocomposite) increases the WCA to 77.4°. The silica-based nanocellulose particles likely introduce surface roughness and reduce the availability of hydrophilic groups at the film–air interface, slightly enhancing water repellency. CS@SiO_2_ reaches 90.1°, crossing the threshold into hydrophobic behavior. This more pronounced increase can be attributed to the intrinsic hydrophobic nature of silica (when not fully hydrated) combined with the creation of a rough surface morphology, which traps air and minimizes water vapor permeability. CS@NC‑SiO_2_ gives the highest WCA of 94.6°. The synergistic combination of bio‑silica (silica-based NC and silica nanoparticles) further amplifies surface roughness and/or alters the surface chemistry, leading to the most hydrophobic film among the tested formulations. The synergistic combination is attributed to electrostatic interactions between chitosan and bio-silica nanoparticles, as well as hydrogen bond interactions among chitosan, nanocellulose, and the Si–OH groups of SiO_2_ [62]. The WCA results are in agreement with the morphology study and barrier properties. From a food packaging perspective, higher water contact angles are desirable for fresh produce preservation, as they reduce surface wettability and can lower moisture adsorption, thereby helping to maintain the firmness and shelf life of products like cherry tomatoes. The progressive increase in WCA across the series suggests that the hybrid nanocomposite (CS@NC‑SiO_2_) offers the best potential as an active packaging material with improved water‑resistant properties [63] .

### Porosity measurements

The textural properties of chitosan biopolymer films influence their adsorption capacity. BET analysis of the film samples (Table 2) shows that the total pore volume and surface area increased for the CS@NC film relative to the pure CS film. This increase is attributed to the nanocellulose (NC) filler, which creates additional pores and enhances the overall porosity of the composite material. Nanocellulose, with its inherent fibrous structure, can introduce internal spaces within the chitosan matrix, leading to a higher accessible surface area for adsorption and other properties [64]. However, the CS@SiO_2_ and CS@NC-SiO_2_ films showed a remarkably lower surface area and pore volume than the other two films (CS and CS@NC). This indicates that the SiO_2_ nanoparticles filled the existing pores well and interacted with the chitosan biopolymer network, subsequently forming an integrated film structure, as examined by SEM surface morphology. Furthermore, the average pore radius was reduced to 1.62, 1.61, and 1.58 nm for the CS@NC, CS@SiO_2_, and CS@NC-SiO_2_ films, respectively. This implies that the nucleating effect of the fillers (bio-silica nanoparticles) was more likely to appear when mixed with chitosan [65]. The total pore volume shows a significant change for the CS@NC-SiO_2_ film compared to the other films, possibly because the NC and SiO_2_ nanoparticles have a strong effect on enclosing the porosity of the host material, leading to a reduction in the average pore volume of the composite and consequently a more tortuous path. This result is in agreement with the WVP and WCA measurements for CS@NC-SiO_2_ with optimal SiO_2_ concentration (Fig. 5(d, e)). This effect can be beneficial for packaging applications [41].

### Light transmittance of chitosan films

**Fig. 6:**
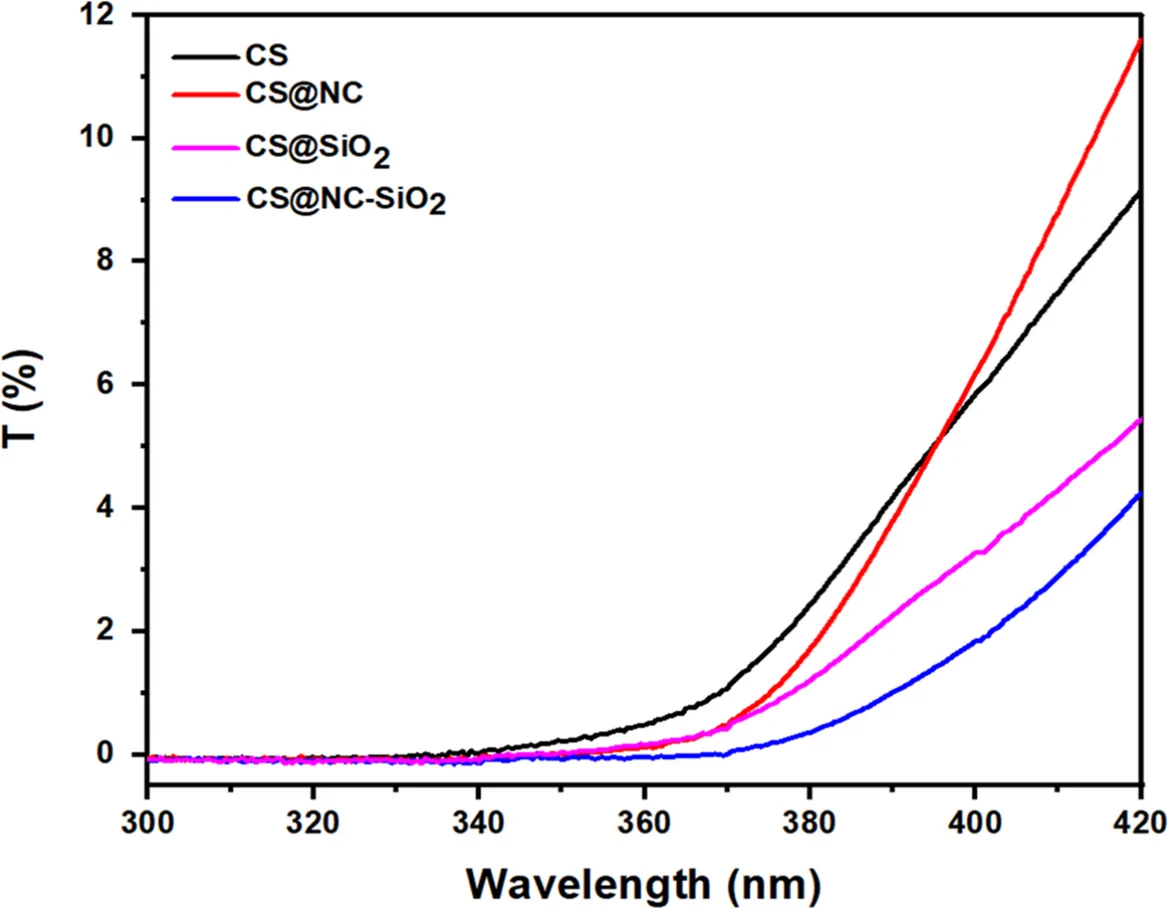
UV- vis spectra of CS, CS@NC, CS@SiO_2_, and CS@NC-SiO_2_ films

One of the most critical properties in the development of food packaging films is their UV–Vis light barrier capacity. This property effectively prevents or delays photo-oxidation of proteins, loss of vitamins, degradation of pigments (such as carotenoids), and lipid oxidation. Therefore, the ability of packaging materials to protect food products from light exposure, especially UV radiation, is highly desirable [66, 67]. The UV–Vis light barrier performance of the fabricated films was assessed by measuring their optical transmittance (%) across wavelengths ranging from 200 to 400 nm. Figure 6 presents the UV-Vis transmittance results for the fabricated films: the control CS film and the composite films CS@NC, CS@SiO_2_, and CS@NS-SiO_2_. All film samples displaying remarkably low transmittance (near zero) in the UV-B region and deep UV (< 280 nm). This indicates excellent blocking of harmful short-wavelength UV radiation. Transmittance remains very low up to approximately 340–360 nm, after which it begins to increase, marking the transition into the UV-A range (320–400 nm). In the UV-A region, the pure CS film shows moderate transmittance (7–9%). Pure chitosan inherently scatters and absorbs UV radiation relatively efficiently owing to its chromophores’ groups (amine, hydroxyl, and acetyl moieties with π bonds or a lone pair of electrons that may absorb the UV) and molecular structure; however, this effect is less pronounced than in the organic/inorganic-enhanced composites. The CS@NC and CS@SiO_2_ films exhibit lower transmittance in the UV-A range (approximately 5–7%) compared to the control CS film. Notably, CS@SiO_2_ demonstrates enhanced UV-blocking performance, most likely due to increased light scattering induced by the inorganic SiO_2_ nanoparticles [68]. The CS@NS-SiO_2_ film achieves the lowest transmittance overall (< 4%), providing the best UV-blocking performance and particularly strong shielding in the UV-A range. This superior performance suggests a synergistic or complementary effect when bio-silica nanoparticles are combined with nanocellulose and chitosan. SiO_2_ nanoparticles contribute strong UV scattering and to some extent absorption due to their high refractive index and surface effects, which create a more tortuous path for UV photons. Meanwhile, nanocellulose likely improves nanoparticle dispersion, enhances film uniformity, and supports better structural integrity. The crosslinking of the chitosan matrix with silica-based NC and SiO_2_ nanoparticles via surface hydroxyl groups may also contribute to improved UV blocking [69]. This crosslinking promotes UV protection through the development of a more rigid and less permeable polymer network, as evidenced by the mechanical and barrier property results. The framework minimizes free volume, chain mobility, and microporosity, as evidenced by porosity measurements, thereby diminishing UV transmission by increasing density and scattering. Furthermore, such a cross-linked network appears resistant to UV damage, ensuring long-term endurance. Furthermore, the transmittance increases noticeably above 400 nm (entering the visible region), confirming that all films maintain reasonable optical transparency in the visible spectrum, which is desirable for many practical applications.

**Table 2:** Characteristics of BET and BJH, UPF rating, and antimicrobial activity of fabricated chitosan films

Sample	CS	CS@NC	CS@SiO_2_	CS@NC-SiO_2_
BET and BJH analysis				
Average Pore radius (nm)	1.6677	1.6200	1.6167	1.5842
Surface Area (m^2^/g)	169.124	238.535	153.902	139.242
Total Pore Volume (cm^3^/g)	0.122789	0.166643	0.104418	0.0948137
UPF rating*				
UPF	39.7	42.8	45.3	57.8
UVA Transmittance %	7.5	7.7	6.8	3.0
UVB Transmittance %	2.5	2.1	2.2	1.8
UPF Rating	Very Good	Excellent	Excellent	Excellent
Protection Grade	II	III	III	III
Antibacterial activity				
Tested pathogens	% of inhibition			
*Escherichia coli* 0157:H7 ATCC 6933 (Gram-negative)	-	-	62.25%	98.12%
*Staphylococcus aureus* ATCC 12,600 (Gram-positive)	-	-	-	61.70%

* Excellent Protection: UPF 40–50^+^, very Good Protection: UPF 25–39, and good Protection: UPF 15–24

UV blocking in this system arises from two distinct mechanisms that can be clearly distinguished from the UPF and transmittance data of the full film series (Table 2; Fig. 6). The chromophore-based absorption is a built-in property associated with chitosan: In neat CS film (UPF = 39.7; UVA = 7.5%; UVB = 2.5%), UV blocking is attributed to amine, hydroxyl, and acetyl groups that absorb radiation in the form of light along with n→π* and π→π* electronic transitions, and it is this photovoltaic absorption that determines its baseline “Very Good” UPF rating [70]. Because this absorption is saturated once all available functional groups have been occupied, it cannot explain the further improvements caused by increasing nanoparticles. The second mechanism is physical light scattering by SiO₂. Because the refractive index of SiO₂ differs from that of the polymer matrix, and because the mesoporous particles present a large internal surface area (TEM, Fig. 1c), incoming UV photons are scattered at numerous nano-scale interfaces. This lengthens the optical path and raises the probability that the light is either absorbed or deflected before exiting the film [71] .

The effect is evident when comparing neat CS with CS@SiO₂: UPF increases from 39.7 to 45.3 while UVA transmittance falls from 7.5% to 6.8%. Since the chemical structure of chitosan remains unchanged, the improvement can be attributed to physical scattering by the SiO₂ particles rather than to any additional chemical absorption. In all four samples, CS (39.7 UPF) represents absorption only. CS@NC (42.8 UPF, 7.7% UVA) exhibits only a slight improvement with a little higher UVA transmittance. CS@SiO₂ (45.3 UPF, 6.8% UVA) exhibits a UPF improvement due to scattering alone. CS@NC-SiO₂ (57.8 UPF, 3.0% UVA, 1.8% UVB) shows the greatest increase over CS, and SiO₂ alone which either mechanism contributes individually, and with the UVA transmittance decreasing more than twofold compared to CS@SiO₂ (6.8%, 3.0%), beyond what is expected from scattering due to SiO₂ loading alone. This synergy originates with NC enhancing SiO₂ dispersion (Fig. 2A(d)), diminishing agglomeration, and augmenting the effective scattering cross-section per unit SiO₂ unit mass (but does not directly induce UV scattering) [72, 28].

### Antibacterial activity

Table 2 shows that under the experimental conditions, pure CS and CS@NC films did not exhibit antibacterial activity against any of the tested bacteria. There are several factors that influence the chitosan activity as an antibacterial agent. These include the medium’s pH, molecular weight, deacetylation degree, bacterial strain type, and growth phase [73]. During neutral conduction, chitosan often shows low inhibition due to inadequate protonation of its amino groups (i.e., the transition of -NH₂ to -NH₃⁺). The CS@SiO_2_ film inhibited *E. coli* by 62.25%, indicating moderate activity. The chitosan antibacterial activity against the tested Gram-negative bacteria was improved by the loading of silica nanoparticles to the film matrix, probably as a result of a better dispersion, a larger contact surface, and a longer contact time with the nanoparticles and bacterial cells [74]. Among all the tested samples, the CS@NC-SiO₂ hybrid nanocomposite film exhibited the best performance with excellent inhibition of E. coli (98.12%) and moderate inhibition of S. aureus (61.70%). This enhanced antibacterial activity can be attributed to the reinforcement of the chitosan matrix by the nanofillers (NC and SiO_2_). These nanofillers not only improve the intrinsic antibacterial properties of chitosan but also reduce bacterial motility and impair biofilm formation, key factors in pathogen survival and virulence. Specifically, SiO_2_ nanoparticles enhance the stability and surface area of the composite, while the NC may improve loading capacity and enable controlled release of active components. Furthermore, the negative charges on the surface of NC (due to abundant hydroxyl groups) and SiO_2_ likely promote greater protonation of the amino groups in chitosan, thereby increasing its positive charge density and antibacterial reactivity, which is a more favorable local environment for maintaining chitosan’s positive charge, facilitating stronger adhesion to the bacterial outer membrane [75].

The differing antibacterial activities observed against *E. coli and S. aureus* can be explained by a single electrostatic interaction acting on two different types of bacterial envelopes. *E. coli*, is a Gram-negative bacterium and has a thin peptidoglycan layer (2–3 nm) surrounded by an outer membrane layer in which the outermost leaflet is composed of lipopolysaccharide (LPS). Phosphate and carboxylate groups in LPS impart a strong net negative surface charge, making the outer membrane highly accessible to cationic species, making the outer membrane a strong electrostatic target. This structural accessibility could elucidate the complete suppression (98.12%) [75] .

*S. aureus*, on the other hand, is Gram-positive and has no outer membrane; rather, it contains a thick (20–80 nm), densely cross-linked peptidoglycan layer with teichoic acids embedded within it. Even though teichoic acids are negatively charged, they are hidden in the peptidoglycan bulk creating steric and diffusional barriers. Therefore, positively charged chitosan particles have to penetrate this matrix in order to come into contact with the cytoplasmic membrane, leading to slower and weaker interactions and a reduced yet still significant amount of inhibition (61.70%) [76].

Mild acidic conditions during the preparation of the film-forming solution (1% acetic acid), the nearly neutral pH of the assay, and that protonate the primary amino groups of chitosan (pKa ~ 6.3–6.5), leading to a mostly cationic (–NH₃⁺) surface in both situations. This cationic surface contributes to the electrostatic binding to anionic constituents of the bacterial envelope (LPS phosphates in *E. coli*, teichoic acids phosphates in *S. aureus*). Sequences of antibacterial activity involve electrostatic interaction, charge-mediated disruption of membranes and cell walls, followed by the release of intracellular contents (proteins, ions, and nucleic acids). In Gram-negative cells, there is additionally enhanced by chitosan chelating divalent cations (Mg²⁺, Ca²⁺) that stabilize the LPS layer; and by chitosan penetrating into the cytoplasm and binding with microbial DNA once inside the cell [77] .

the introduction of NC and SiO₂ does not represent a new antibacterial mechanism rather, it amplifies the already active electrostatic one. The anionic functional groups of NC (sulfate esters) and SiO₂ (SiO⁻) raise the surface negative charge density of the film, leading to even more protonation of the amines of chitosan at the film bacterium interface, which results in a higher positive charge density in the film available for electrostatic attack. This is why neither pure CS nor CS@NC films exhibited any inhibition in viability (Table 2). In the absence of SiO₂, the cationic charge density is unable to surpass the threshold required for antibacterial efficacy in these assay conditions. Physical contact with the bacterial surface is additionally improved by SiO₂ as well as bacterial localized membrane depolarization. These results are in agreement with previous reports stating the predictably enhanced susceptibility of Gram-negative bacteria in comparison to Gram-positive bacteria towards chitosan-based materials due to relatively easy penetration through the anionic outer membrane opposed to the steric hindrance created by thick peptidoglycan layer found in Gram-positive cells [63, 64]. This performance underscores the strong potential of the CS@NC-SiO₂ film as an active material for food packaging applications.

### Cherry tomatoes packaging

Fresh fruits contain high levels of water and sugars, making them very vulnerable to microbial spoilage after harvest, which is a major factor in postharvest deterioration [78]. Cherry tomatoes *(Solanum lycopersicum)* are widely appreciated for their high nutritional content and pleasant taste, making them one of the most consumed and versatile fruits worldwide. However, their short shelf life and high metabolic activity after harvesting significantly limit their storage duration and commercial value [79]. To evaluate the real-world effectiveness of the newly developed chitosan@bio-silica nanocomposite films in food packaging, fresh cherry tomatoes were wrapped using these films. Over a 21-day storage period at 4 °C, the physicochemical and microbiological changes in the packaged tomatoes were measured.

**Fig. 7:**
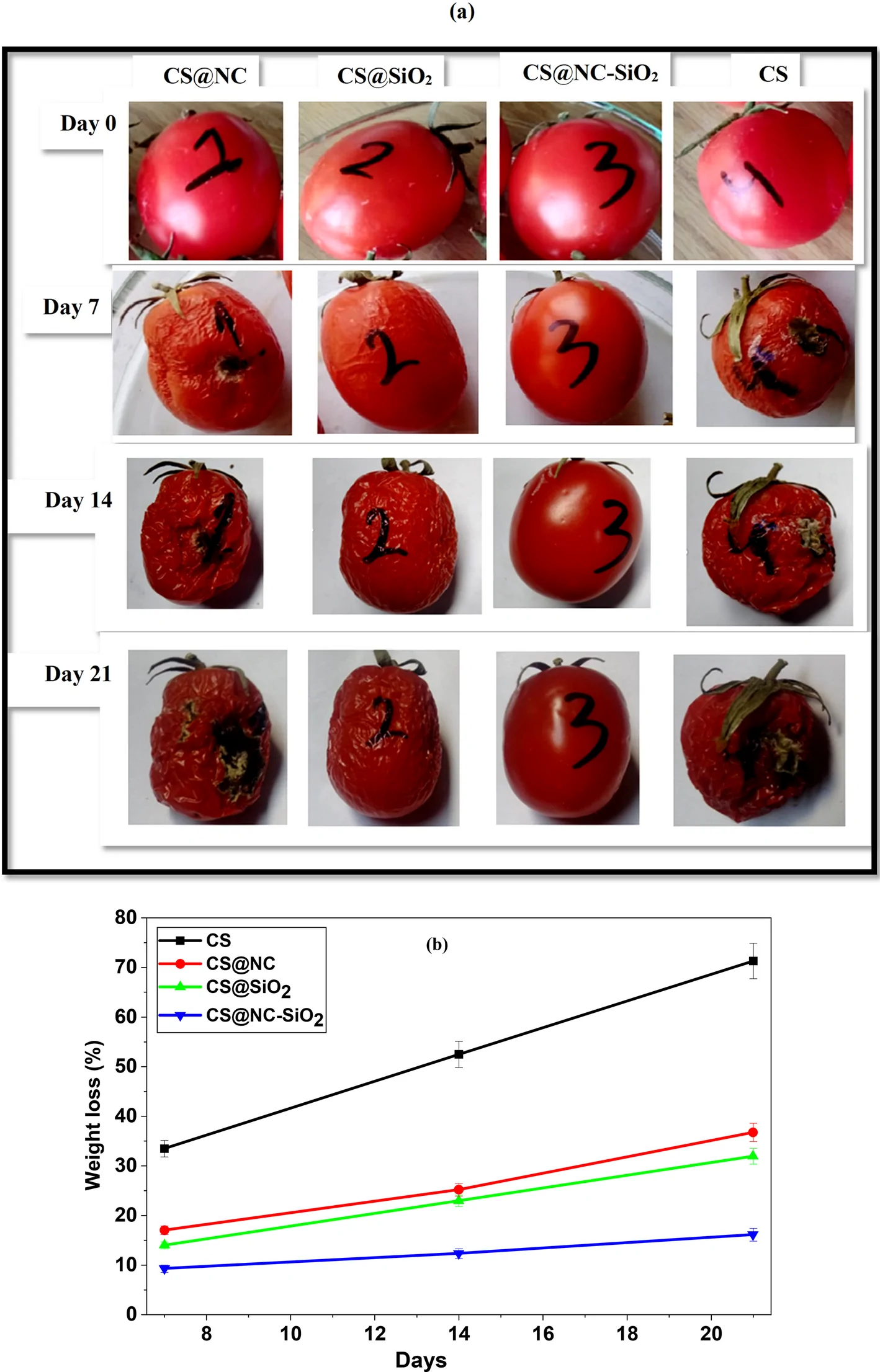
Digital images of cherry tomatoes **a** stored at 4°C for 21 days, and changes in the weight loss % **b** of the tomatoes packaged with the chitosan nanocomposite films during storage

**Fig. 8:**
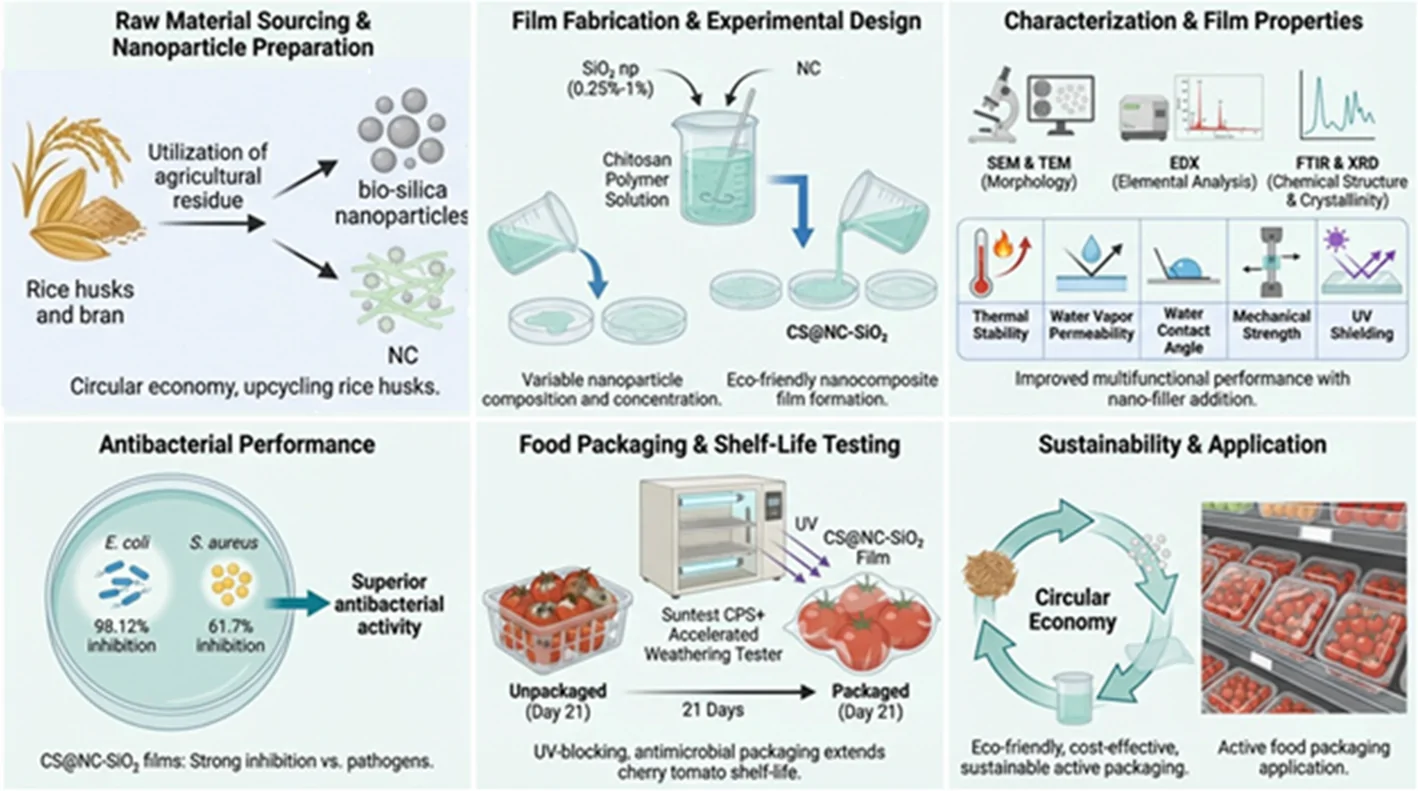
Graphical representation of the sustainable UV-shielding CS@NC-SiO_2_ nanocomposite film

Figure 7(a) shows that the four treatment groups exhibited different degrees of fruit decay over time. Across all groups, weight loss of cherry tomatoes steadily increased during storage (Fig. 7(b)). This trend was largely due to ongoing respiration and transpiration processes. Additionally, the limited ability of the packaging films to block water vapor also contributed to moisture loss from the fruit [80]. The experiment explores the post-harvest preservation of cherry tomatoes wrapped in four distinct films, designated 1 through 4, after being subjected to intense accelerated UV aging and then stored in a refrigerator for 3 weeks. Week-by-Week observation as the following:

Week 1 (Day 7): At this early stage, changes were already apparent. CS@NC-SiO2 (S3) remained completely stiff and shiny while experiencing just a slight weight reduction of 9.28%. Other samples showed minimal wrinkling and a loss of around 17–33%. With weight reductions of 33.47% and 17.02%, respectively, the control CS and CS@NC samples were the most impacted, and they had already shown signs of moderate mold development and weak regions. Day 14 of Week 2: There was an obvious performance disparity. CS@NC-SiO2(S3) remained almost intact and had the least amount of loss (12.32%). CS@NC-SiO2(S3) remained practically intact and suffered the least amount of loss (12.32%). CS@SiO2 (S2) lost 22.95% and remained reasonably satisfactory. In contrast, clean CS (S4) got excessively wrinkled, lost around half of its initial value, and finally went moldy and decomposed. Week 3, Day 21: The final ranking was immediately discernible in terms of weight and look. S3 is still red, plump, and marketable, with a measly 16.12% total loss. S2 is wrinkled, but remains satisfactory after weight loss (31.74%). Other samples (S1 and S4) were significantly shriveled and partially rotten, with losses ranging from 37 to 71%. Only S3 (and S2) remained economically viable after three weeks. Over the course of 21 days, the film applied on S3 offered outstanding and consistent protection against photo-oxidative degradation, impeded gas exchange (i.e., shielding barrier), and perhaps antimicrobial benefits (preventing fast dehydration and fungal assault on tomato surfaces). Even when the tomatoes were purposely challenged with exceptionally high UV doses, moisture loss was greatly reduced, and mold development and tissue collapse were almost completely eliminated. Based on these findings, it can be concluded that of the four packaging films tested, the one used on S3 is by far the most effective at extending the shelf life of cherry tomatoes under severe UV conditions and refrigerated storage, retaining both appearance and more than 83% of their original weight after three weeks. This means strong moisture barrier, UV blocking, and antibacterial properties. This synergistic effect demonstrated that the chitosan bio silica nanocomposite films successfully protected the natural quality of cherry tomatoes during storage. Overall, the created nanocomposite film has promise for enhancing food safety by actively managing the surrounding food environment, perhaps reducing reliance on synthetic preservatives and chemicals. The CS@NC SiO₂ nanocomposite film can serve as an active packaging material for a range of food products, providing a valuable route for high value applications of biomass materials while meeting the demand for environmentally friendly and sustainable solutions [81]. These results also confirmed that the CS film modified with silica-based NC and SiO₂ nanoparticles effectively inhibits fruit decay. Figures (8) provides a visual representation of. Figures (8) presents a graphical illustration of the sustainable UV-shielding CS@NC-SiO_2_ nanocomposite film.

It is important to note that this work reveals the potential of using chitosan@bio-silica nanocomposite films for eco-friendly active packaging. There are some limitations that we need to consider. A comparative study between citric acid and other pretreatment protocols such as HCl, H₂SO₄, NaOH for bio-silica isolation is still required. The evaluation of the packaging performance should be conducted on more types of fruit under different storage conditions, and the antimicrobial activity should be expanded and investigated with other relevant pathogens. Toxicity evaluation and adherence to food-contact material regulations should be addressed, together with direct comparison to commercial packaging materials. Biodegradation property and cost-effectiveness have also yet to be analyzed. These limitations provide a clear path forward for future work, including further formulation development and optimization, testing against a broader range of microbes and food matrices, and a full safety assessment and evaluation vis-à-vis existing commercial options.

## Conclusion

Every year, vast quantities of agro-waste are generated worldwide. Recycling these residues through valorization techniques offers substantial economic and environmental benefits. In this study, sustainable biopolymer-based composites were developed as eco-friendly active food packaging materials. Chitosan-based nanocomposite films functionalized with bio-silica nanoparticles (NC and/or SiO₂) were successfully fabricated and thoroughly evaluated. Rice husk residues served as a renewable source for producing silica-based nanocellulose (NC) via acid hydrolysis. Pure silica nanostructures (SiO₂) were also extracted from the same residues using citric acid pretreatment followed by calcination at 600 °C. Morphological analyses, combined with FTIR and XRD data, confirmed that the NC@SiO₂ particles were uniformly dispersed and well-integrated within the chitosan matrix. The incorporation of NC@SiO₂ significantly enhanced the films’ UV-blocking capacity and thermal stability in a concentration-dependent manner. Among all formulations, the CS@NC-SiO₂ films exhibited the best overall performance, with superior mechanical properties including a tensile strength of 25.37 MPa, elongation at break of 27%, and Young’s modulus of 593 MPa. These films also demonstrated excellent UV protection (UPF ≈ 58, with UVA and UVB transmittance of 3% and 1.8%, respectively), increased surface hydrophobicity, and strong antibacterial activity achieving 98% inhibition against *Escherichia coli* and 61.70% against *Staphylococcus aureus*. Practical packaging trials using cherry tomatoes confirmed that the CS@NC-SiO₂ films effectively preserved fruit quality, maintained weight stability, and extended shelf life up to 21 days under UV light exposure. Overall, these findings highlight the potential of CS@NC-SiO₂ films as a multifunctional, sustainable solution for advanced active food packaging. By valorizing rice husk waste, this approach not only prolongs food freshness through combined antimicrobial and barrier properties but also reduces environmental impact and minimizes economic losses due to postharvest spoilage.

## Data Availability

The datasets generated during and/or analysed during the current study are available from the corresponding author on reasonable request.
